# The Role of HER2 and RANK in Breast Cancer and New Therapeutic Approaches With Denosumab, Anti‐HER2 Antibodies and Immunotherapy

**DOI:** 10.1111/jcmm.71071

**Published:** 2026-02-23

**Authors:** Panagiotis Sarantis, Aristofania Simatou, Ioanna A. Anastasiou, Kostas Palamaris, Eleni‐Myrto Trifylli, Evangelos Koustas, Christina Piperi, Michalis V. Karamouzis, Athanasios G. Papavassiliou

**Affiliations:** ^1^ Department of Biological Chemistry, Medical School National and Kapodistrian University of Athens Athens Greece; ^2^ Department of Pharmacology, Medical School National and Kapodistrian University of Athens Athens Greece; ^3^ Department of Pathology, Medical School National and Kapodistrian University of Athens Athens Greece; ^4^ Second Department of Internal Medicine—GI‐Liver Unit, General Hospital ‘Hippokratio’, Medical School National and Kapodistrian University of Athens Athens Greece; ^5^ Oncology Department General Hospital ‘Evangelismos’ Athens Greece; ^6^ Academic Department of Internal Medicine, General and Oncology Hospital ‘Agioi Anargyroi’ National and Kapoditrian University of Athens Athens Greece

**Keywords:** breast cancer, Denosumab, HER2, immune checkpoint inhibitors, Pertuzumab, RANK, RANKL, Trastuzumab

## Abstract

Despite advances in targeted therapies, resistance to anti‐human epidermal growth factor receptor 2 (HER2) treatments remains a significant challenge in breast cancer (BC) management. This study aimed to evaluate the effectiveness of a triple‐targeting regimen—Denosumab (D), Pertuzumab (P) and Trastuzumab (T)—for HER2‐positive (HER2^+^) BC, and to assess the added value of immune checkpoint inhibitors (ICIs). Immunohistochemical analysis of 120 paraffin‐embedded BC samples revealed that HER2^+^ tumours exhibited significantly higher receptor activator of nuclear factor (NF)‐κB (RANK) expression compared to HER2‐negative (HER2^−^) tumours. Notably, RANK‐positive (RANK^+^)/HER2^+^ patients who received triple‐targeting therapy experienced a statistically significant improvement in disease‐free survival (DFS), while RANK‐negative (RANK^−^)/HER2^+^ patients did not derive similar benefit. In BT‐474 HER2^+^ xenograft mouse models, the combination of D+P+T significantly reduced tumour volume and weight. Additional analyses showed elevated *signal transducer and activator of transcription 3* (*STAT3*) expression in HER2^−^ tissues and higher *mechanistic target of rapamycin* (*mTOR*) expression in HER2^−^ compared to HER2^+^ samples. Importantly, three‐dimensional (3D) cell culture experiments demonstrated that adding ICIs (Nivolumab (N) and Ipilimumab (I)) to the triple‐targeting regimen further reduced cell viability in HER2^+^ BC cells. These results underscore the pivotal role of the RANK–receptor activator of NF‐κB ligand (RANKL) axis in tumour growth and immune regulation, supporting the use of triple‐targeting therapy and suggesting enhanced benefits with the inclusion of ICIs to potentially overcome therapeutic resistance in HER2^+^ BC.

## 1 Introduction

Breast cancer (BC) is one of the most common malignancies encountered in the female population [1]. However, it is characterised by significant heterogeneity, encompassing distinct histological subtypes defined by the expression of specific biomarkers, including oestrogen receptor (ER), progesterone receptor (PR) and human epidermal growth factor receptor 2 (HER2) [2]. Among these, HER2‐positive (HER2^+^) tumours are of particular interest, accounting for approximately 20% of all BCs and historically associated with poor clinical outcomes [3]. Nevertheless, recent evidence indicates improved prognosis in this subgroup, largely due to the introduction of HER2‐targeted therapies [4]. Trastuzumab represents the most prominent agent in this therapeutic class and has significantly reshaped both the treatment strategy and prognosis of patients with HER2^+^ BC [4]. Despite these advances, the increasing incidence of resistance to currently available anti‐HER2 therapies highlights the urgent need to explore alternative therapeutic approaches [5]. Ideally, such novel agents would be used in combination with existing therapeutic strategies to overcome resistance and further enhance their anticancer efficacy. In this context, the receptor activator of nuclear factor (NF)‐κB (RANK) and its ligand, receptor activator of NF‐κB ligand (RANKL), have been recently shown to be involved in all hallmarks of BC [6, 7, 8, 9]. Furthermore, the inhibition of the RANK–RANKL axis has proven anticancer activity, and it is suggested to be implicated in the reversal of resistance in HER2^+^ tumours, with our previous in vitro studies demonstrating that the combined administration of anti‐HER2 and anti‐RANKL agents further induces tumour reduction [10, 11].

Moreover, the RANK–RANKL axis has been shown to promote the development of an immunosuppressive tumour microenvironment (TME) by facilitating infiltration of protumorigenic neutrophils and macrophages while limiting CD8^+^ T‐cell recruitment [12, 13]. Consistent with this, combination therapy using a RANKL inhibitor (Denosumab) and immune checkpoint inhibitors (ICIs) has been associated with a more pronounced attenuation of tumour growth [12]. Meanwhile, it is well established that BC is generally considered a moderately immunogenic neoplasm exhibiting a ‘cold’ phenotype [14]. Notably, HER2^+^ tumours are associated with higher tumour mutational burden (ΤΜΒ) [15, 16]. Consequently, large ongoing randomised clinical trials are currently evaluating the combination of anti‐HER2 agents with ICIs, aiming to overcome drug resistance and convert immunologically ‘cold’ tumours into a ‘hotter’ phenotype [15, 16].

Herein, we further validated our previous findings [10, 11] in vivo through a retrospective analysis of the efficacy of triple‐targeting therapy in formalin‐fixed paraffin‐embedded samples from patients with HER2^+^ BC exhibiting immunohistochemical expression of the RANK–RANKL axis. Furthermore, we provide, for the first time, evidence from three‐dimensional (3D) cell line models demonstrating a strong association between the RANK–RANKL axis and immunomodulation in HER2^+^ BC. Specifically, we show that, beyond triple targeting (Denosumab (D)+Pertuzumab (P)+Trastuzumab (T)), the addition of ICIs (anti‐programmed death receptor 1 (PD‐1) and anti‐cytotoxic T‐lymphocyte‐associated protein 4 (CTLA‐4); Nivolumab (N) and Ipilimumab (I), respectively) leads to further tumour reduction in HER2^+^ BC through enhanced immunostimulation and the release of peripheral immune tolerance.

## 2 Materials and Methods

### 2.1 Patient Samples

Between January 2020 and July 2024, paraffin‐embedded tissue blocks were obtained from 120 patients diagnosed with early‐stage, locally advanced or metastatic BC. Of these cases, 75/120 were HER2^+^ and 45/120 were HER2‐negative (HER2^−^), with a median age at diagnosis of 56.5 years. During the same period, fresh tumour specimens were additionally collected from surgical resections of 12 patients (6 HER2^+^ and 6 HER2^−^). Adjacent non‐tumorous tissue was not included in the analysis. Written informed consent was obtained from all participants, and the study was conducted under approval of the National and Kapodistrian University of Athens Medical School Ethics Committee (protocol number 195/13.12.2019).

### 2.2 Immunochemistry

Formalin‐fixed, paraffin‐embedded sections from 120 human BCs were sliced at 4‐μm thickness. Tissue sections were deparaffinised at 62°C for 30 min, then in 3 consecutive xylene buffers for 3 min each. Rehydration was performed through a graded series of ethanol solutions (100%, 96%, 80% and 70%), after which sections were rinsed with distilled water (dH_2_O). Antigen retrieval was carried out by heating the sections in citrate buffer (pH 6.0) at 95°C for 20 min. Endogenous peroxidase activity was subsequently blocked by incubation with 3% H_2_O_2_ (v/v) in dH_2_O for 10 min at room temperature (RT), and sections were then washed with phosphate‐buffered saline (PBS) and blocked with 5% normal goat serum (NGS) for 1 h. Thereafter, sections were incubated overnight with a polyclonal rabbit anti‐RANK primary antibody (sc‐9072, Santa Cruz Biotechnology, Dallas, TX, USA; 1:100). Following PBS washes, sections were incubated with biotinylated secondary antibodies (cat. no. 20775, Merck Millipore, Burlington, MA, USA) for 10 min at RT, followed by incubation with streptavidin‐horseradish peroxidase (HRP) (cat. no. 20774, Merck Millipore, Burlington, MA, USA) for an additional 10 min. Immunoreactivity was visualised using 3,3′‐diaminobenzidine (DAB). Haematoxylin solution (Sigma‐Aldrich, St. Louis, MO, USA) was used for counterstaining. Sections were then dehydrated through graded ethanol solutions (70%, 80%, 96% and 100%) and mounted with mounting medium onto glass coverslips. Immunostaining was evaluated using a histoscore (H‐score), calculated by multiplying the percentage of positively stained neoplastic cells by staining intensity.

### 2.3 BC Xenografts

BT‐474 HER2^+^ BC xenografts were established by subcutaneous injection of 5 × 10^6^ cells into the flanks of female immunodeficient (NOD/SCID) mice aged 6–8 weeks. Cells were resuspended in Corning Matrigel Basement Membrane Matrix and injected in accordance with the manufacturer's instructions (CLS354234, Corning Inc., Corning, NY, USA). Tumour growth was monitored by calliper measurements, and tumour volume was calculated using the formula: Volume = ½ × Length × Width^2^. Once a palpable tumour was detected (25 days after the initial cell inoculation), mice were randomised into three treatment groups (*n* = 5 per group): (a) control, receiving no therapeutic intervention, (b) T+P, treated with Trastuzumab (10 mg/kg) and Pertuzumab (10 mg/kg), administered intraperitoneally (i.p.) twice weekly and (c) T+P+D treated with Trastuzumab (10 mg/kg), Pertuzumab (10 mg/kg) and Denosumab (10 mg/kg), administered i.p. twice weekly. Treatments continued for 15 days. At the experimental endpoint, mice were euthanised, and tumours were excised, weighed and photographed. Individual tumour weights were recorded, and representative tumour specimens were imaged for qualitative comparison.

### 2.4 RNA Extraction—cDNA Synthesis

Twelve fresh samples were obtained from 12 patients immediately after surgery, immersed in RNAlater solution (Ambion, Austin, TX, USA) and stored at 4°C for subsequent RNA extraction. In total, 100–200 mg of tissue was utilised from each sample. Total RNA was extracted using the RNeasy Mini Kit (Qiagen, Hilden, Germany), according to the manufacturer's instructions. RNA was quantified using a Thermo Scientific NanoDrop Lite Spectrophotometer (Thermo Scientific, Waltham, MA, USA), and RNA purity was evaluated based on the A260/A280 ratio. Samples with an A260/A280 ratio of approximately 2.0 were used for complementary DNA (cDNA) synthesis, while cDNA was synthesised using the PrimeScript RT Reagent Kit (Takara Bio, Kusatsu, Shiga, Japan) following the manufacturer's protocol.

### 2.5 Quantitative Real‐Time Polymerase Chain Reaction (qRT‐PCR)

qRT‐PCR reactions were performed using cDNA along with KAPA SYBR FAST qPCR Master Mix (2×) Kit (KK4602, Sigma‐Aldrich, St. Louis, MO, USA), gene‐specific forward and reverse primers and nuclease‐free water. All genes of interest for all samples were analysed simultaneously, together with *glyceraldehyde 3‐phosphate dehydrogenase* (*GAPDH*), which served as a universally expressed housekeeping control gene. Relative gene expression levels were calculated using the comparative Ct (2^−^ΔΔCt) method, with normalisation to *GAPDH* expression. Primer sequences used in this study are summarised in Table 1.

**TABLE 1:** Primer sequences.

Gene	Primer type	Sequence
*STAT3*	Forward primer	GAAACAGTTGGGACCCCTGA
	Reverse primer	AAGCGGCTATACTGCTGGTC
*mTOR*	Forward primer	CGCGAACCTCAGGGCAAG
	Reverse primer	TGGTTTCCTCATTCCGGCTC
*GAPDH*	Forward primer	GTCTCCTCTGACTTCAACAGCG
	Reverse primer	ACCACCCTGTTGCTGTAGCCAA

### 2.6 Cell Culture

Peripheral blood mononuclear cells (PBMCs) were isolated from peripheral blood collected from healthy donors in ethylene diamine tetraacetic acid (EDTA)‐containing tubes. Whole blood was layered over a density gradient medium (Ficoll‐Paque) and centrifuged at 300–400 × *g* for 30 min at RT. The PBMC layer, located above the density gradient, was carefully collected and washed twice with PBS to remove any residual plasma and gradient medium. The BC cell lines used in this study included MCF‐7 and SK‐BR‐3, which were cultured in Dulbecco's Modified Eagle's Medium (DMEM, Thermo Fisher Scientific, Waltham, MA, USA) supplemented with 10% heat‐inactivated fetal bovine serum (FBS, Sigma‐Aldrich Co., St. Louis, MO, USA) and 1% of a mixture of penicillin–streptomycin (1000 U/mL–10 mg/mL, Thermo Scientific, Fremont, CA, USA). All cell lines were obtained from either the American Type Culture Collection (ATCC) or the European Collection of Authenticated Cell Cultures (ECACC).

### 2.7 3D Co‐Culture of PBMCs and Cancer Cells

For 3D co‐culture experiments, a low‐attachment culture plate was pre‐coated and matrix components were mixed according to the desired co‐culture conditions. PBMCs and cancer cells were combined in a tube, at a starting ratio of 1:2 (cancer cells to PBMCs). The cell suspension was mixed with Matrigel according to the manufacturer's instructions, typically at a ratio of 50% cell suspension to 50% matrix. The resulting mixture was then immediately added to the wells of the culture plate (approximately 100 μL per well) prior to the solidification of the matrix. The plates were incubated at 37°C in a humidified atmosphere containing 5% CO_2_ to allow the culture solidification, which occurred within 30–60 min. Subsequently, an appropriate volume of culture media (10%–20% of the total volume) was added to each well without disturbing the matrix. Media changes were conducted every 2–3 days, ensuring that the matrix remained continuously hydrated throughout the culture period.

### 2.8 Cell Viability Measurement

Cell viability was assessed using the MTT assay in 96‐well plates, where six biological replicates of 3D cell culture cells were seeded as previously described. Following treatment, the reagent 3‐(4,5‐dimethylthiazol‐2‐yl)‐2,5‐diphenyltetrazolium bromide (MTT) (30006, Biotium Inc., Landing Parkway, Fremont, CA, USA) was added to each well at a final concentration of 0.5 mg/mL. The cells were incubated with MTT for 4 h at 37°C in a 5% humidified CO_2_ atmosphere. The MTT assay relies on the ability of metabolically active cells to convert the yellow tetrazolium salt MTT into purple formazan crystals. Following incubation, the formazan precipitate was dissolved in dimethyl sulfoxide (DMSO) and the colour intensity was quantified by measuring absorbance at 570 nm using a Multiskan FC Microplate Photometer (Thermo Scientific, Fremont, CA, USA). Measure background absorbance at 630 nm was removed from signal absorbance to obtain normalised absorbance values (optical density (OD)). Cell viability (%) = (experimental group OD—zero adjustment group OD)/(control group OD—zero adjustment group OD) × 100%. Absorbance measurements were performed with a Multiskan FC Microplate Photometer (Thermo Scientific, Fremont, CA, USA).

### 2.9 Statistical Analyses

Statistical calculations and diagrams were performed using the statistical software SPSS V25 (IBM, New York, NY, USA) and Graph‐Pad Prism (V5.0., San Diego, CA, USA). Bivariate Pearson and Spearman Correlations, Student *t*‐test, Kaplan–Meier Survival Analysis with log‐rank testing, Chi‐Square analysis and ANOVA were performed followed by Dunnett's T3 post hoc test. All results, with a two‐sided *p* level ≤ 0.05, were considered statistically significant.

## 3 Results

### 3.1 Immunohistochemical Analysis of RANK Expression in HER2 ^+^ and HER2 ^−^ BC Samples

**FIGURE 1:**
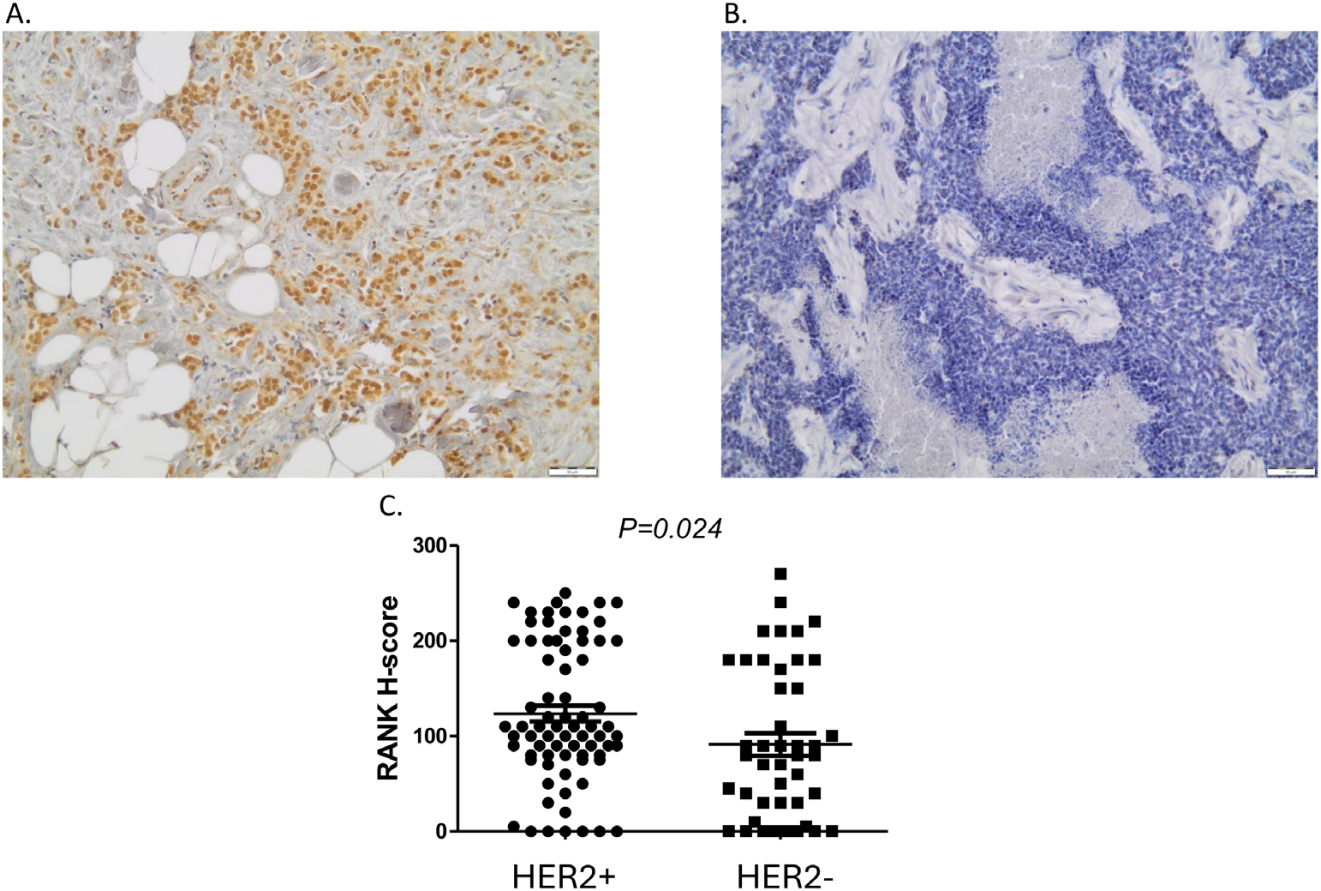
RANK expression in HER2^+^ and HER2^−^ tissue samples. Representative immunohistochemical staining of tissue sections demonstrating differential RANK expression. The left panel (A) displays strong, widespread brown staining indicative of elevated RANK levels, while the right panel (B) shows a lack of staining. The bottom panel (C) presents a quantitative comparison of RANK H‐scores between HER2^+^ and HER2^−^ samples. HER2^+^ tissues exhibit significantly higher RANK expression (mean H‐score) than their HER2^−^ counterparts (*p* = 0.024), suggesting a potential correlation between HER2 status and RANK pathway activation. Magnification ×200.

First, we performed immunochemistry with RANK antibody in 120 formalin‐fixed, paraffin‐embedded samples to compare the expression of RANK between HER2^+^ and HER2^−^ BC samples. There was a statistically significant difference in the expression of RANK between HER2 groups (higher expression in HER2^+^ patients, *p* = 0.024). The mean RANK H‐score for patients with HER2^+^ status is 123.7, while the mean value for patients with HER2^−^ status is 91.3 (Figure 1A–C).

### 3.2 Enhanced Tumour Suppression With Triple Combination Therapy in BT‐474 Xenograft Model

**FIGURE 2:**
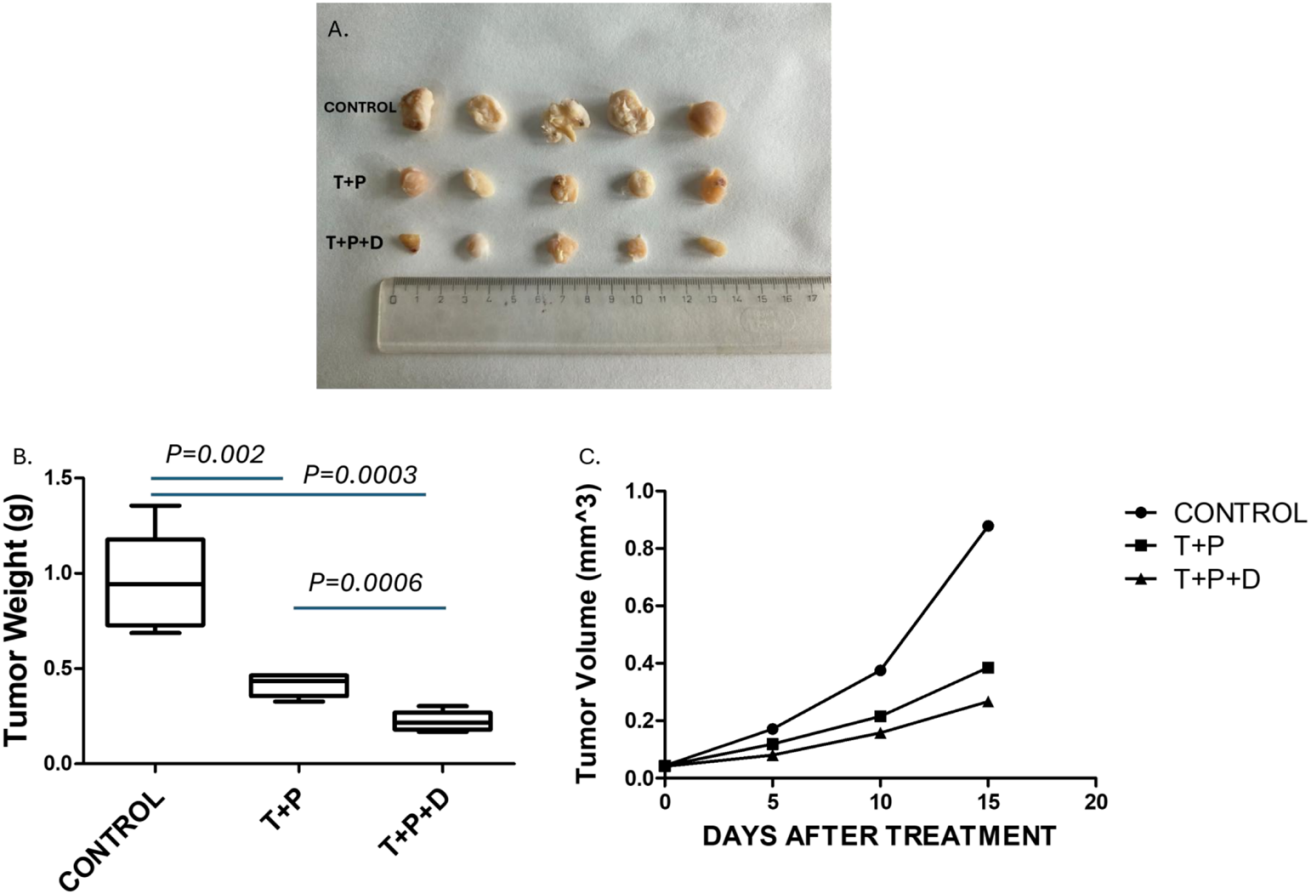
Tumour growth in BT‐474 breast cancer (BC) xenografts under different treatment conditions. (A) Representative images of tumours excised from immunodeficient mice bearing subcutaneous BT‐474 HER2^+^ BC xenografts. Mice were treated according to the following regimens: Control (no treatment), T+P (Trastuzumab+Pertuzumab) and T+P+D (Trastuzumab+Pertuzumab+Denosumab). The control group exhibited large, well‐formed tumours with minimal variation. The T+P group displayed a moderate reduction in tumour volume and increased heterogeneity, consistent with dual HER2 blockade. The T+P+D group demonstrated the most pronounced tumour suppression, with markedly smaller and irregular masses, suggesting synergistic effects of RANKL inhibition in combination with HER2 targeted therapy. (B) Tumour weight at the protocol endpoint. The T+P combination significantly reduced tumour weight compared with Control (*p* = 0.002), while the triple treatment T+P+D produced an even greater reduction (*p* = 0.0003 vs. Control; *p* = 0.0006 vs. T+P). (C) Tumour growth curves. The T+P+D regimen resulted in the strongest inhibition of tumour progression relative to all other groups.

Next, we wanted to study the action of Denosumab (D) in combination with dual HER2 blockade in an in vivo model. Treatment of BT‐474 xenografts with Trastuzumab (T) and Pertuzumab (P), either alone or in combination with D, resulted in significant reductions in both tumour volume and tumour weight compared to the control group. Mean tumour volume decreased from 0.878 cm^3^ in the control group to 0.385 cm^3^ in the T+P group (−56.15%), and further to 0.266 cm^3^ in the T+P+D group (−69.70%). The addition of D to the dual HER2 blockade yielded an additional 30.91% reduction in volume compared to T+P alone (Figure 2A,B).

Consistent with these findings, tumour weight measurements showed a marked decline across treatment groups. Mean tumour weight in the control group was 0.9516 g, while the T+P group averaged 0.4146 g (−56.43%) and the T+P+D group 0.2224 g (−76.63%). These reductions were statistically significant (*p* < 0.01), indicating enhanced antitumor efficacy with the triple combination therapy (Figure 2A,C).

### 3.3 Impact of Denosumab and Triple Therapy on Disease‐Free Survival (DFS) in BC Subgroups Defined by HER2 and RANK Expression and Hormone Receptor Interplay

**FIGURE 3:**
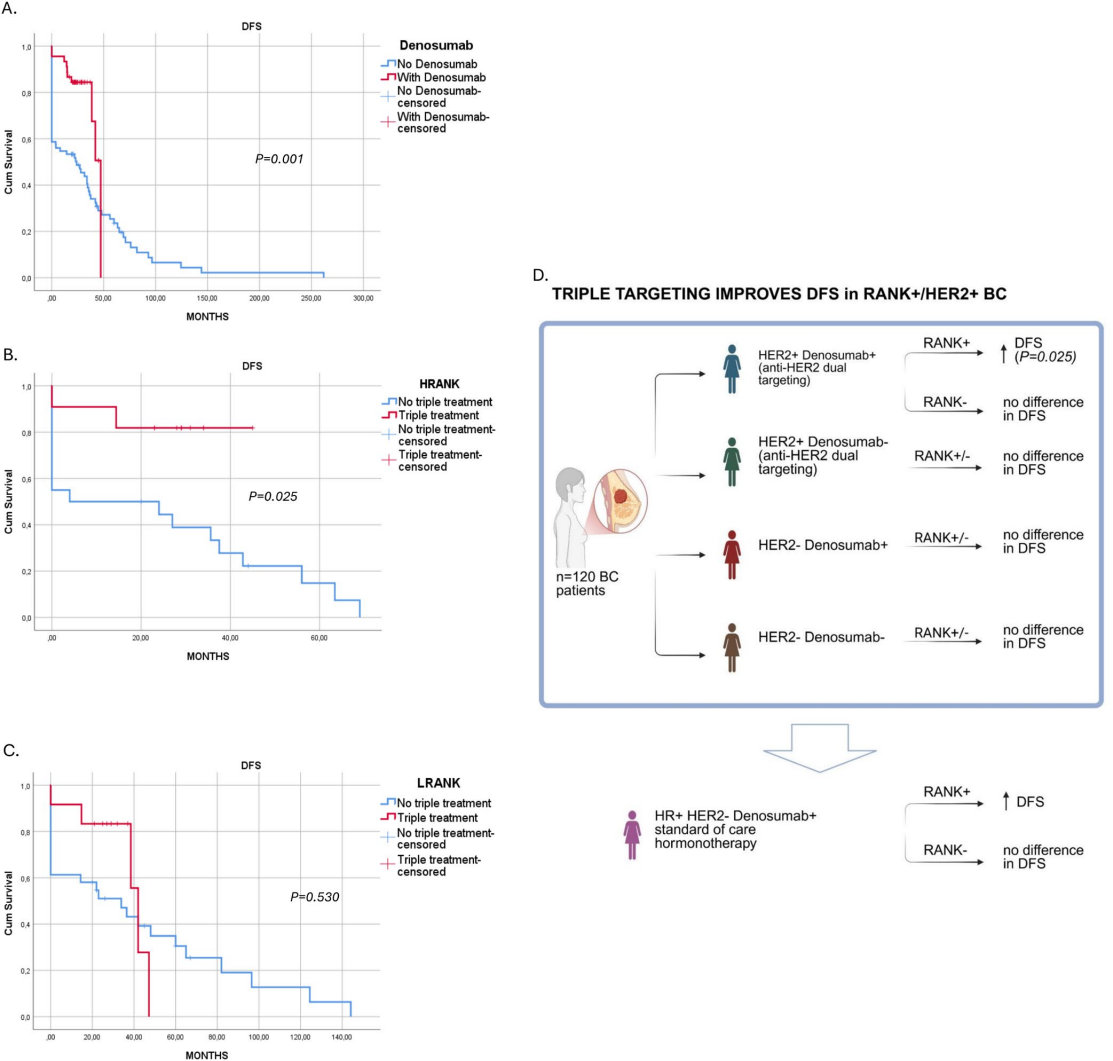
Kaplan–Meier curves depicting disease‐free survival (DFS) across treatment groups. Kaplan–Meier survival analysis was performed to evaluate DFS among patients receiving different therapeutic interventions. (A) DFS comparison between patients treated with Denosumab (red line) and those not receiving Denosumab (blue line). (B) DFS comparison between patients with high RANK (HRANK) expression receiving triple treatment (red line) and those without triple treatment (blue line). (C) DFS comparison between patients with low RANK (LRANK) expression receiving triple treatment (red line) versus no triple treatment (blue line). Censored observations are indicated by ‘+’ symbols. (D) Schematic presentation of the above results: Following immunohistochemical detection of RANK expression in 120 paraffin‐embedded blocks from breast cancer (BC) patients, RANK^+^HER2^+^ patients who received triple targeting (T+P+D) had a statistically significant benefit in DFS (*p* = 0.025) compared to RANK^−^HER2^+^ BC (*p* = 0.530) (created using BioRender (https://biorender.com/)). HR+, hormone receptor‐positive.

Having observed a therapeutic benefit from the triple‐combination treatment in the in vivo model, we next determined whether similar patterns are evident in BC patients with distinct molecular profiles. Patients with independent HER2 status who were treated with Denosumab demonstrated significantly improved DFS, with a *p*‐value of 0.001 (Figure 3A). This suggests a strong statistical correlation between D treatment and enhanced DFS outcomes in these patients. Patients displaying high RANK expression who underwent triple therapy showed a significantly improved DFS, with a *p*‐value of 0.025 (Figure 3B). This finding highlights the potential impact of high RANK expression on treatment outcomes, suggesting that the combination of triple therapy could be particularly beneficial for this subgroup of patients. The findings are further supported by the observation that patients with reduced RANK expression who received triple therapy exhibited no statistically significant difference in DFS (*p* = 0.530) (Figure 3C). This underscores the potential implication of RANK expression levels in treatment efficacy and disease outcomes in this patient cohort (Figure 3D).

Further analysis of the clinicopathological and molecular characteristics of patients revealed several statistically significant findings. Notably, co‐expression patterns of progesterone receptor (PR) and oestrogen receptor (ER) were identified, with a Chi‐Square analysis yielding a *p*‐value of < 0.001, indicating a highly significant association between the two receptors. Additionally, the relationship between HER2 status and PR expression was explored, with a *p*‐value of 0.002 demonstrating an inverse correlation; specifically, when HER2 expression is higher, PR expression is lower and vice versa. A similar trend was observed concerning HER2 status and ER expression, although this correlation did not reach statistical significance (*p* = 0.090). These findings provide important insights into the interplay between hormone receptors and HER2 status.

### 3.4 Distinct Expression Patterns of mTOR and STAT3 in HER2 Different Status in Breast Tumours Identified by qRT‐PCR Analysis

**FIGURE 4:**
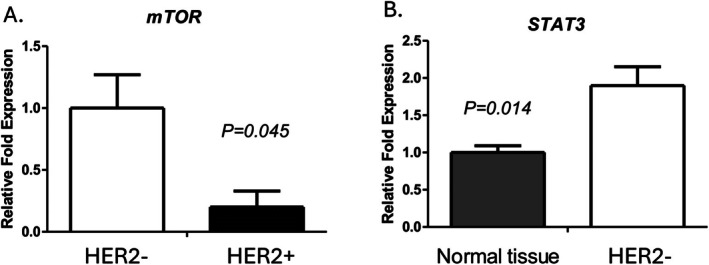
Differential expression of *mTOR* and *STAT3* in HER2‐associated breast tissue samples. Bar graphs depict the relative fold expression of *mTOR* and *STAT3* in normal and tumour tissues stratified by HER2 status. (A) *mTOR* expression is markedly higher in HER2^−^ samples relative to their HER2^+^ counterparts, suggesting differential pathway activation based on HER2 status. (B) *STAT3* expression is significantly elevated in HER2^−^ tumour tissue compared to normal tissue, indicating a potential involvement in tumorigenic signalling pathways. Error bars represent standard deviation across biological replicates. Data are normalised to control expression levels.

qRT‐PCR analyses were then performed with fresh patient samples (HER2^+^, HER2^−^ and normal tissue) to determine the role of HER2 in important pathways. The following genes were analysed: *mechanistic target of rapamycin* (*mTOR*) and *signal transducer and activator of transcription 3* (*STAT3*). A 5‐fold increase in *mTOR* expression in HER2^−^ versus HER2^+^ tumours (Figure 4A) and a 2‐fold increase in *STAT3* expression in HER2^−^ tumours compared to normal tissue was found (Figure 4B). Statistical analysis showed that there is a statistically significant decrease in the *mTOR* gene in HER2^+^ tumours (*p* = 0.045), while in HER2^−^ tumours there is a statistically significant increase in the *STAT3* gene (*p* = 0.014) (Figure 4A,B).

### 3.5 Synergistic Reduction of Cell Viability in 3D BC Co‐Culture Model by Single and Combination Drug Therapies

**FIGURE 5:**
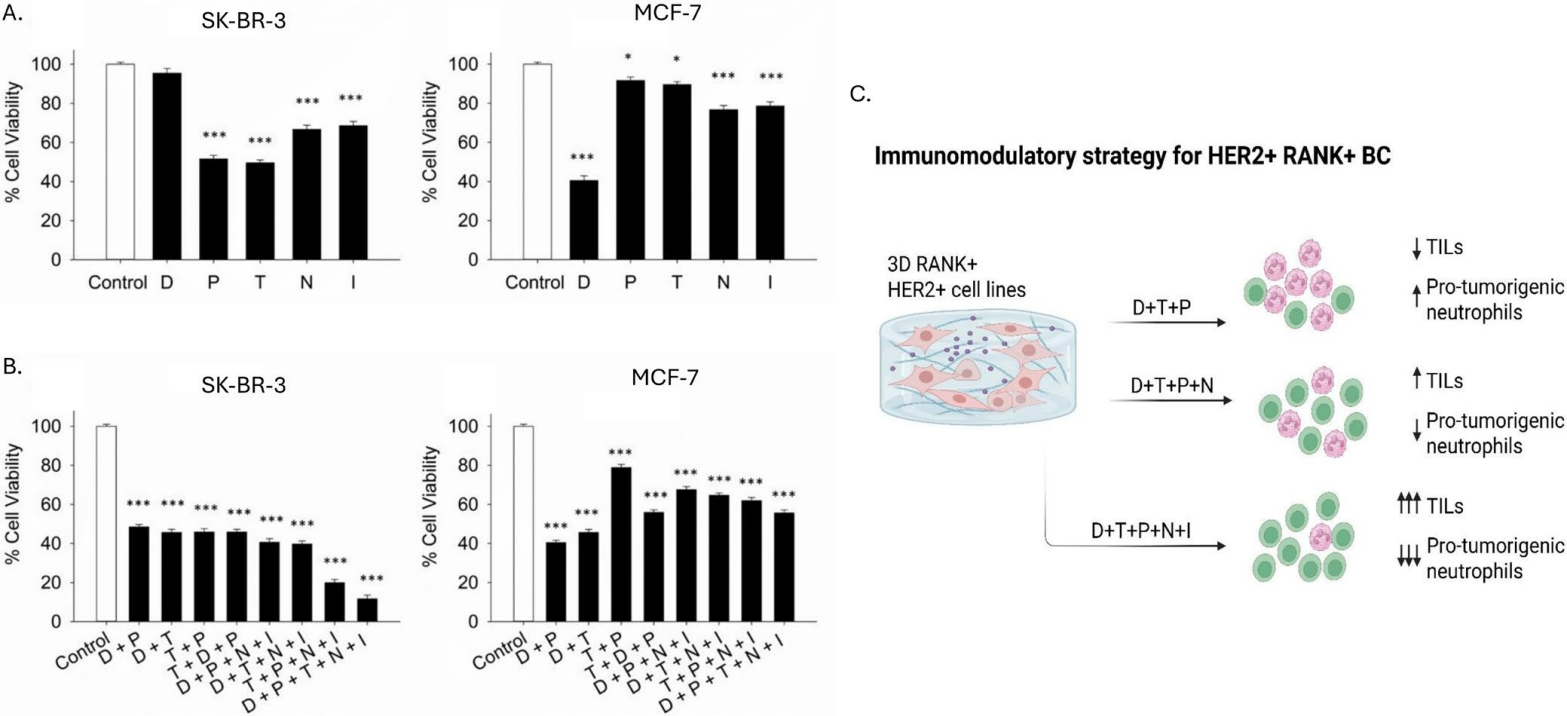
Cell viability levels of SK‐BR‐3 and MCF‐7 determined by MTT assay. (A, B) Effects of single (A) and in combination (B) therapeutic agents on cell viability in HER2^+^ (SK‐BR‐3) and HER2^−^ (MCF‐7) breast cancer (BC) cell lines. The data is expressed as mean ± SD of three independent experiments (*N* = 3). Statistical significance was determined by ANOVA followed by Dunnett's T3 post hoc test, where *p‐value < 0.05; **p‐value < 0.01; ***p‐value < 0.001 in comparison with the control group. (C) Experiments at the level of 3D cell lines, when incubated with D+T+P+N+I (Denosumab+Trastuzumab+Pertuzumab+Nivolumab+Ipilimumab) in HER2^+^ BC, resulted in a clinically significant reduction in cell viability, possibly through immunomodulation of the tumour microenvironment (TME), characterised by a marked increase in tumour‐infiltrating lymphocytes (TILs) infiltration and a possibly concurrent decrease in pro‐tumorigenic neutrophils (created using BioRender (https://biorender.com/)). ANOVA, analysis of variance; MTT, 3‐(4,5‐dimethylthiazol‐2‐yl)‐2,5‐diphenyltetrazolium bromide; SD, standard deviation.

Finally, we studied the effect of the addition of ICIs to the above tested drugs (D, P and T) in HER2^+^ and HER2^−^ cell lines. In SK‐BR‐3 cells, treatments with drugs D, T, P and the ICIs anti‐PD‐1 Nivolumab (N) and anti‐CTLA‐4 Ipilimumab (I) each resulted in a substantial reduction in viability, ranging from approximately 40% to 60% (except D) compared to the control group, with reductions reaching statistical significance at *p* < 0.001 (Figure 5A; denoted by the asterisks). This indicates that these single agents possess potent cytotoxic activity against SK‐BR‐3 cells. Remarkably, drugs N and I demonstrated the most pronounced effects, with reductions exceeding 55%, underscoring their greater efficacy within this context. Similarly, in MCF‐7 cells, the same single‐drug treatments led to significant decreases in viability, with lower reductions compared to SK‐BR‐3 cells. Only D shows a greater reduction in viability, and this probably indicates that HER2^−^ is more RANKL‐dependent. Importantly, the combination treatment comprising D+P+T+N+I exhibited a synergistic or at least additive effect, resulting in a reduction in cell viability in both cell lines, which significantly surpassed the effects observed with individual drugs (*p* < 0.001) (Figure 5B). This marked enhancement highlights the potential therapeutic advantage of multi‐drug strategies to reduce drug resistance and improve overall efficacy. Moreover, overall analysis indicates that SK‐BR‐3 cells are consistently more sensitive to these treatments, with greater decreases in viability observed across all conditions, compared to MCF‐7 cells.

## 4 Discussion

It is known that the RANK‐RANKL axis is involved through its main mediator, NF‐κB, in all hallmarks of cancer [6, 7, 8, 9] and in immune modulation [17, 18, 19, 20] in different cancer subtypes. More specifically, recent studies show that the RANK pathway is implicated in hormone receptor‐positive (HR^+^) BC [21], triple‐negative BC (TNBC) [22] and breast cancer gene 1 (BRCA1)‐associated BC [23, 24, 25]. Additionally, the latest scientific reports elucidate its crucial role in tumour progression and the development of resistance to anti‐HER2 agents in HER2^+^ BCs [10]. In this study, we examined and revealed a putative triple‐targeting strategy in RANK^+^HER2^+^ BCs, which could also be extended to a quintuple targeting strategy in an attempt to immunomodulate these tumours. The physical association between RANK and HER2 members has been verified in a previous study by performing proximity ligation assay (PLA) [10]. In HER2^+^ BC cell lines (BT‐474, SK‐BR‐3), a high incidence of RANK:HER2 heterodimers was observed, which was notably decreased in ER^+^HER2^−^ BCs, suggesting a functional correlation between HER2:RANK dimer formation and HER2 protein expression. As expected, RANKL addition promotes dimer formation, in contrast to Denosumab and anti‐HER2 agents, which strongly disrupt dimer formation. Employing functional experiments (MTT and wound recovery), it was also shown that triple targeting (D+T+P) reduced both proliferative and migratory potential in SK‐BR‐3 BCs [10].

To determine the important role of HER2, we performed qRT‐PCR on fresh tissues of patients who were positive or negative for HER2. The increased expression of *mTOR* in HER2^−^ compared to HER2^+^ breast tumours can be attributed to differences in the phosphoinositide 3‐kinase (PI3K)/AKT/mTOR signalling pathway. The increased expression of *mTOR* in HER2^−^ tumours may be due to alternative activation mechanisms, such as PI3K mutations or phosphatase and tensin homologue (PTEN) loss, which drive uncontrolled pathway signalling. Furthermore, HER2^−^ tumours may depend on mTOR signalling for survival, particularly in cases of chemotherapy resistance [26, 27, 28].

The 2‐fold increase of *STAT3* expression in HER2^−^ breast tumours compared to normal tissue can be explained by several factors. First, STAT3 is a transcription factor that is often activated in many types of cancers, including BC. In HER2^−^ BCs, which do not overexpress the HER2 protein, other signalling pathways such as the STAT3 pathway may become upregulated to compensate for the lack of HER2 signalling. Moreover, the TME in HER2^−^ tumours may also influence *STAT3* expression. Factors like cytokines and growth factors produced by surrounding stromal cells can activate STAT3. Overall, the increased *STAT3* expression in HER2^−^ breast tumours may indicate a potential therapeutic target in managing this subtype of BC [29, 30, 31, 32].

The present study demonstrates that triple combination therapy with Denosumab, Trastuzumab and Pertuzumab (D+T+P) yields enhanced antitumor efficacy compared to dual HER2 blockade (T+P) or untreated controls in the BT‐474 HER2^+^ BC xenograft model. The observed reductions in both tumour volume and tumour weight underscore the therapeutic potential of combining RANK inhibition into established HER2‐targeted regimens. Previous studies have implicated RANK/RANKL signalling in BC progression, particularly in the context of HR^+^ and HER2‐enriched subtypes. The enhanced efficacy observed in the D+T+P group aligns with emerging evidence that RANK inhibition may sensitise tumours to antibody‐based therapies by disrupting stromal support and immune evasion mechanisms [33, 34].

Data from recent preclinical studies have shown that RANK signalling in mouse mammary tumour cells exerts an immunosuppressive environment by preventing CD8 T‐cell recruitment and promoting the infiltration of pro‐tumorigenic neutrophils [12, 18]. These findings are corroborated by the D‐BEYOND trial, which evaluated the effect of Denosumab on tumour immune infiltration in 24 premenopausal women with early BC. Administration of two doses of Denosumab resulted in a significant increase in tumour‐infiltrating lymphocytes (TILs), particularly T‐cells and CD8 T‐cells. Patients were classified as ‘responders’ (≥ 10% increase in TILs) or ‘non‐responders.’ Identified biomarkers of response included elevated T‐regulatory cell infiltration, serum RANKL levels and tumour RANK activation [12]. Additionally, the D‐BIOMARK study further confirmed that Denosumab, when used in the neoadjuvant setting, significantly enhanced TILs levels, suggesting its crucial role in regulation of tumour‐immune crosstalk [17]. Therefore, the rationale for co‐administration of ICIs with Denosumab is now strongly supported, in an effort to enhance immune stimulation [13, 18]. Currently, several phase 3 trials such as ASTEFANIA, APTneo and Impassion 050 are examining the combination of anti‐HER2 agents with ICIs toward converting the ‘cold’ immune phenotype of HER2^+^ early BC into a ‘hot’ one [14].

Based on the above and given the synergistic effect demonstrated between Denosumab and anti‐HER2 agents, we proceeded with the next series of experiments to assess the benefit of adding immunotherapy to the triple combination described so far. Therefore, 3D cell cultures were conducted adding single (anti‐PD‐1 (Nivolumab)) or dual (Nivolumab+anti‐CTLA‐4 (Ipilimumab)) ICIs. More specifically, the subgroup exposed to quintuple targeting (D+T+P+N+I) demonstrated both a numerically and statistically significant tumour reduction compared to the triple (D+T+P) or even quadruple (D+T+P+N) targeting, highlighting a promising immunomodulatory strategy for HER2^+^ BC (Figure 5C).

Finally, we also collected 120 human paraffin‐embedded BC tissues of patients with early, locally advanced or metastatic BC. Among these patients, 75/120 were HER2^+^ and 45/120 were HER2^−^. We retrospectively evaluated the effectiveness of the combination therapy (D+T+P) in this cohort of patients in relation to the immunohistochemical expression of RANK. Initially, we confirmed that HER2^+^ patients had higher RANK expression as previously shown [35]. Interestingly, the staining pattern of RANK was almost exclusively nuclear. We also observed that patients treated with Denosumab had a statistically significant longer DFS (*p* = 0.005). Then we evaluated the possible correlation between high RANK expression levels and DFS outcomes. Remarkably, patients with high RANK expression levels who received triple targeting (D+T+P) had a statistically significant benefit in DFS (*p* = 0.025). When we focused on patients with low expression of RANK receiving triple targeting, no difference in DFS was observed (*p* = 0.530). Based on the above, we suggest that RANK could serve not only as a prognostic [22, 35], but also as an important predictive biomarker especially for HER2^+^ BCs. Additionally, we demonstrated that HR^+^RANK^+^ patients who received dual targeting with Denosumab and standard of care hormonotherapy also faced a benefit in DFS. This is in agreement with the findings of previous preclinical studies suggesting that continuous exposure of RANK^+^HR^+^ cell lines to RANKL induces HR loss and resistance to hormone therapy through a feedback loop mechanism [21]. Regarding nuclear localisation of RANK, it has been reported in tumour cells of osteosarcoma, while in BC it is observed for the first time. Nuclear relocalisation of membrane receptors has been documented for other oncogenic drivers, including epidermal growth factor receptor (EGFR) and HER2, where it is associated with transcriptional regulation of proliferation, survival and DNA repair genes. Such a mechanism provides a plausible explanation for the enhanced aggressiveness and therapeutic resistance observed in RANK^+^HER2^−^ enriched tumours [36].

Therefore, we demonstrated for the first time in in vivo experiments that triple targeting in RANK^+^HER2^+^ BCs is associated with an increase in DFS. Noteworthy, two phase 3 randomised clinical trials (ABCSG‐18 and D‐CARE) have been conducted regarding the potential benefit of adding Denosumab to standard of care therapy in patients with early BC, revealing conflicting results [37, 38]. According to the results of the 8‐year long term analysis of ABCSG‐18, Denosumab improves DFS by 3.5% in postmenopausal women with HR^+^ BC [39]. In agreement with the findings of the aforementioned study, we demonstrated a statistically significant benefit in DFS in the overall population receiving Denosumab. On the other hand, in the D‐CARE study, Denosumab failed to show benefit in DFS and did not improve disease‐related outcomes for women with high‐risk early BC (stage II, III). However, we should point out that in the D‐CARE study, only about 65% of patients were HR^+^ and they included patients who were generally at increased risk for the development of distant metastases and not specifically for bone metastases [37]. The inability to demonstrate a DFS benefit in this study may be attributed to the absence of appropriate patient selection, given that, according to our findings, the observed benefit seems to be primarily driven by the HER2^+^/RANK^+^ subpopulation. Lastly, neither the GeparX study, which examined the addition of Denosumab to the neoadjuvant setting, was able to show a benefit in the pathologic complete response (pCR) rate, which was the primary endpoint of this study [40]. Although there were numerically fewer distant relapses with Denosumab, this did not reach statistical significance. Collectively, these observations provided further support to our hypothesis that a meaningful DFS benefit emerges when Denosumab is administered in the adjuvant rather than in the neoadjuvant setting.

Based on the data described so far, we can assume the significant clinical importance of triple targeting with D+T+P in RANK^+^HER2^+^ subpopulation which leads to an increase in DFS, suggesting that RANK could function as a predictive biomarker, while at the level of cell lines additional benefits are observed with the implementation of ICIs beyond the triple combination.

In conclusion, this study confirms that human samples from patients with early, locally advanced or metastatic BC (RANK^+^HER2^+^), where triple targeting (D+T+P) was found to be associated with a statistically significant benefit in DFS. The noticeable antitumor effect, at the clinical level, of Denosumab when combined with anti‐HER2 agents such as Trastuzumab and Pertuzumab in HER2^+^RANK^+^ patients raises the possibility for therapeutic strategies with existing drugs in order to overcome the induced resistance in a specific BC subpopulation. To date, there is no study examining the antitumor activity of Denosumab in patients with HER2^+^ BC. Our in vivo findings strongly encourage the need for setting up new clinical trials in larger patient populations to evaluate whether dual blockade of RANK^+^HER2^+^ patients, either with triplet (D+T+P) or through newer anti‐HER2 agents such as Trastuzumab‐Deruxtecan in combination with Denosumab, would lead to significant advantages in DFS and overall survival (OS). Given the synergistic action of Denosumab + anti‐HER2 agents, we demonstrated for the first time the potential immunomodulatory effect of Denosumab when co‐administered with ICIs, particularly in HER2^+^ cancers that display intrinsic resistance to immunotherapy. At the level of 3D cell lines, it was observed that the quintuple targeting (D+T+P+N+I) in HER2^+^ BCs led to a statistically significant reduction in tumour size compared to the other combinations. The results from the qRT‐PCR show extra molecular targets and the use of inhibitors, this time for HER2^−^ BC patients. Consequently, it is considered essential to conduct further studies at the level of clinical trials to evaluate the benefits of anti‐HER2, anti‐RANK, ICIs co‐administration in an effort to overcome immune resistance.

## Author Contributions

P.S. and A.S. have substantially contributed to the conception and design of the study, performance of the experiments, data acquisition, analysis, and interpretation of data and drafting the manuscript. I.A.A., K.P., E.‐M.T., E.K. and C.P. have substantially contributed to the analysis and interpretation of data. M.V.K. and A.G.P. have substantially contributed to the conception and design of the work, performance of the experiments, analysis and interpretation of data, supervision and drafting and revising the manuscript. All authors have approved the final version of the manuscript.

## Funding

The authors have nothing to report.

## Ethics Statement

This study protocol was reviewed and approved by the Ethics Committees of the Medical School of the National and Kapodistrian University of Athens and the ‘Agios Savvas’ Oncology Hospital of Athens. Formalin‐fixed paraffin‐embedded and fresh tissue samples were collected immediately after surgery at ‘Agios Savvas’ Hospital. All patients were fully informed and provided written consent for the use of their tissues for analysis and for the publication of the results in scientific journals. The study was also approved by the National and Kapodistrian University of Athens Medical School Ethics Committee (protocol number 195/13.12.2019), covering the use of both human samples and experimental animals.

## Conflicts of Interest

The authors declare no conflicts of interest.

## Data Availability

The data that support the findings of this study are available from the corresponding authors upon reasonable request.
